# Household Coverage of Fortified Staple Food Commodities in Rajasthan, India

**DOI:** 10.1371/journal.pone.0163176

**Published:** 2016-10-19

**Authors:** Grant J. Aaron, Prahlad R. Sodani, Rajan Sankar, John Fairhurst, Katja Siling, Ernest Guevarra, Alison Norris, Mark Myatt

**Affiliations:** 1 Global Alliance for Improved Nutrition (GAIN), Geneva, Switzerland; 2 Indian Institute of Health Management Research, Jaipur, Rajasthan, India; 3 Valid International, Oxford, United Kingdom; 4 Brixton Health, Llawryglyn, Wales, United Kingdom; The Hospital for Sick Children, CANADA

## Abstract

A spatially representative statewide survey was conducted in Rajasthan, India to assess household coverage of atta wheat flour, edible oil, and salt. An even distribution of primary sampling units were selected based on their proximity to centroids on a hexagonal grid laid over the survey area. A sample of *n* = 18 households from each of *m =* 252 primary sampling units PSUs was taken. Demographic data on all members of these households were collected, and a broader dataset was collected about a single caregiver and a child in the first 2 years of life. Data were collected on demographic and socioeconomic status; education; housing conditions; recent infant and child mortality; water, sanitation, and hygiene practices; food security; child health; infant and young child feeding practices; maternal dietary diversity; coverage of fortified staples; and maternal and child anthropometry. Data were collected from 4,627 households and the same number of caregiver/child pairs. Atta wheat flour was widely consumed across the state (83%); however, only about 7% of the atta wheat flour was classified as fortifiable, and only about 6% was actually fortified (mostly inadequately). For oil, almost 90% of edible oil consumed by households in the survey was classified as fortifiable, but only about 24% was fortified. For salt, coverage was high, with almost 85% of households using fortified salt and 66% of households using adequately fortified salt. Iodized salt coverage was also high; however, rural and poor population groups were less likely to be reached by the intervention. Voluntary fortification of atta wheat flour and edible oil lacked sufficient industry consolidation to cover significant portions of the population. It is crucial that appropriate delivery channels are utilized to effectively deliver essential micronutrients to at-risk population groups. Government distribution systems are likely the best means to accomplish this goal.

## Introduction

Food fortification is defined here as deliberately increasing the content of one or more essential micronutrients to commonly consumed foods so as to provide public health benefits [1]. The micronutrients are delivered by “piggybacking” on the consumption of these foods, which are known as “vehicles.” Provided that a selected vehicle is commonly consumed (i.e., a staple food in the diet), mass or large-scale fortification can be a highly cost-effective approach to reaching a large proportion of a population without the need to change dietary behaviors [1,2]. Many countries, including India, have implemented food fortification strategies as a means to increase micronutrient intakes in the diet.

The success of such programs depends on the simultaneous presence of a number of factors, which are described in detail in the World Health Organization (WHO) guidelines on fortification [1]. Briefly, the following are essential:

- The vehicle should be universally, or nearly universally, consumed by members of the target population. If no single vehicle is universally consumed, then fortification of more than one vehicle may be required to achieve high coverage.
- There should be a large degree of consolidation/centralization in the production, processing, and distribution of the vehicle to allow cost-effective fortification and quality control.
- The fortification package (i.e., the micronutrients) should address an identified micronutrient need in the target population.
- The fortificants must be bioavailable, safe, and have favorable organoleptic properties (i.e., should not lead to the development of off-colors or off-flavors).
- The fortification levels should be informed by the consumption patterns (i.e., quantity and frequency of consumption of the vehicle) in the program population.
- Enabling legislation and regulations should be present and there should exist a political, financial, and scientific consensus to sustain both initial and recurring fortification costs.

Legislation relating to food fortification has been in place in India for more than 60 years [3,4]. Most recently, the government recommended fortification in its 10th (2002), 11th (2007), and 12th (2012) 5-year plans for government-led nutrition programs [4]. Aside from iodization of salt and fortification of “Vanispati Ghee” (a low-cost ghee substitute) with vitamin A, fortification is devolved to individual states within the country’s federal system.

In 2010, the Global Alliance for Improved Nutrition (GAIN) started a voluntary fortification initiative in the states of Madhya Pradesh and Rajasthan [5]. These states were selected based on elevated prevalences of key nutritional indicators (stunting in children, wasting in children, low birth weight, low body mass index in women, and anemia in women and children) relative to national averages and the presence of both government and industry support [5]. This article focuses on the Rajasthan program. Program activities are summarized in Table 1.

**Table 1:** Activity Summary for the GAIN Rajasthan Fortification Program^a^.

Start date	Vehicle	Micronutrient	Fortification	Units
			level	
		Iron (FeSO_4_)	30	ppm
February 2012	Atta wheat flour	Folic acid	1.3	ppm
		Vitamin B12	0.01	ppm
November 2012	Edible oils	Vitamin A (retinyl palmitate)	25,000	IU kg^-1^
		Vitamin D2	2,000	IU kg^-1^
June 2013	Milk	Vitamin A (retinyl acetate)	2,000	IU L^-1^
		Vitamin D2	400	IU L^-1^

^a^ppm, parts per million; IU, international units; kg, kilograms; L, liters

Rajasthan participates in the Indian Universal Salt Iodization program [6] and, until November 2013, distributed atta wheat flour fortified with iron and folic acid targeted at poorer households through its public distribution system (PDS).

The main objective of the work reported here was to assess statewide household coverage of the fortified staple food commodities in Rajasthan. All programs (voluntary and government-led) were assessed. All food vehicles except milk were assessed.

## Materials and Methods

### Survey and sample design

The survey was designed to be spatially representative of the state. An even distribution of primary sampling units (PSUs) (i.e., villages/city blocks) was selected from across the state. This approach is suited to assessing coverage over wide areas where there is a need to detect and map heterogeneity of coverage [7,8]. PSUs (i.e. villages/city blocks) were selected based on their proximity to centroids of a hexagonal grid laid over the survey area. The resulting sample is a triangular irregular network [8,9]. A variable intensity sampling design was used [9]. In rural areas, the sample density was such that no person lived more than about 32 kilometers from a sampling point. Sampling density increased with increasing population density. A sample of *n* = 18 households from each of *m* = 252 PSUs was taken. The within-community sample in villages used systematic sampling of dwellings in the villages (or parts of villages) organized as a ribbon (or ribbons) of dwellings, and a random walk “EPI3” sampling strategy in villages (or parts of the villages) organized as clusters of dwellings [10]. For urban areas, selected PSUs were divided into blocks; four blocks in each PSU were selected at random. All households within the selected blocks were sampled. This sample design provides *implicit stratification*, selecting a sample that is distributed across both the entire survey area and within sampled communities, and tends to spread the sample among important subgroups of the population—e.g., rural, urban, and peri-urban areas; administrative areas; ethnic/religious sub-populations; and socioeconomic groups—and often improves the precision of estimates made from survey data [11,12]. Households were defined as one person living alone or a group of people (not necessarily related to each other) living at the same address with common housekeeping (sharing either a living room or a sitting room or at least one meal each day) [13]. To be eligible for the survey the household must have contained a caregiver with a child aged under 2 years old. The caregiver may have been the child’s biological mother or the person who cared for and gave the child most meals on most days. If a caregiver had more than one eligible child, then the oldest eligible child was selected as the youngest eligible child would be likely to be aged below 6 months and should be exclusively breastfed. Among households selected for the survey, data on all people living in the household were collected using a household roster. These data were limited to age, sex, and educational history. A broader dataset was collected about a single caregiver / child pair selected from within each household.

#### Ethical clearance and survey administration procedures

Ethical clearance to conduct the survey was obtained from the Indian Institute of Health Management Research Institutional Committee for Ethics and Review of Research (IIHMR). Oral consent to participate was obtained from the child’s principal caregiver on the basis that participation in the survey was voluntary. Written consent was not sought due to concerns regarding the adult female literacy rate in Rajasthan. Consent was recorded on the survey questionnaire. This process was approved by the IIHMR. Data were collected by trained interviewers supervised by experienced field supervisors. Data were collected using paper forms and reviewed daily by field supervisors for consistency, ranges, and legal values. Paper forms were transported to a centralized unit for double-entry and validation using the CSPro data-entry and checking system (version 5.03).

### Survey instrument

Data were collected on demographic and socioeconomic status; education levels of household members; housing conditions; recent infant and child mortality; water, sanitation, and hygiene practices; food security; child health; infant and young child feeding (IYCF) practices; maternal dietary diversity; coverage of fortified staples; and maternal and child anthropometry. All survey modules (i.e., question and indicator sets) were taken from validated guidelines with language, wording, and layout finalized through pilot testing in the field. All case-definitions (e.g., for maternal and child undernutrition, hunger, poor sanitation, suboptimal IYCF practices) adhered to internationally recognized standards. Staple food coverage question sets and indicators were adapted from a pilot survey conducted previously [14]. Each food vehicle was assessed in a separate module. Atta wheat flour had two modules: one module assessed the voluntary fortification program, and the other module assessed Atta wheat flour distributed through the PDS system.

#### Indicators of risk

**Fig 1:**
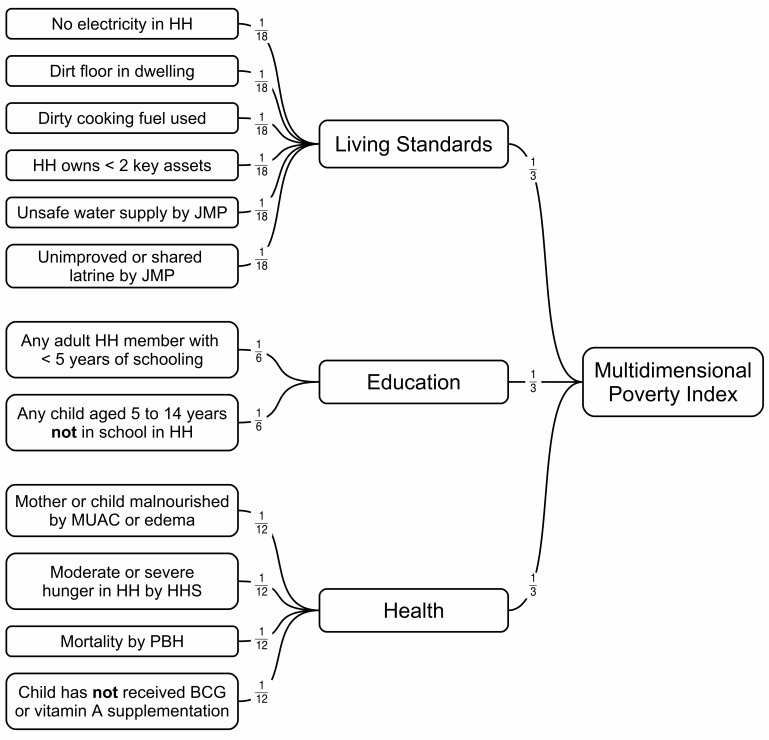
Component indicators and weightings used to calculate the MPI. HH = household; JMP = WHO/UNICEF Joint Monitoring Program for Water Supply and Sanitation; MUAC = mid-upper arm circumference; HHS = Household Hunger Score; PBH = previous birth history; BCG = Bacillus Calmette-Guérin vaccine

Four indicators of risk were assessed to investigate the degree to which the fortified staple foods met population needs. The risk indicators were poverty, poor (i.e., below median) maternal dietary diversity, sub-optimal IYCF practices, and rural residence. Poverty was assessed using the Multidimensional Poverty Index (MPI) as shown in Fig 1 [15]. Maternal dietary diversity was assessed using the Woman’s Dietary Diversity Score (WDDS) [16]. IYCF practices were assessed using the Infant and Child Feeding Index (ICFI) [17]. Further details of these indicators are published elsewhere [14]. Rural residence was determined by reference to the census data used to draw the survey sample. Note that coverage is assessed at the household level and that individual risk factors are analyzed at the household level. If a household contained a child with sub-optimal IYCF practices, then the household is classified as at-risk by sub-optimal IYCF. If the interviewed caregiver reported below median dietary diversity, then the household is classified as at-risk by low WDDS.

### Indicators of coverage

**Fig 2:**
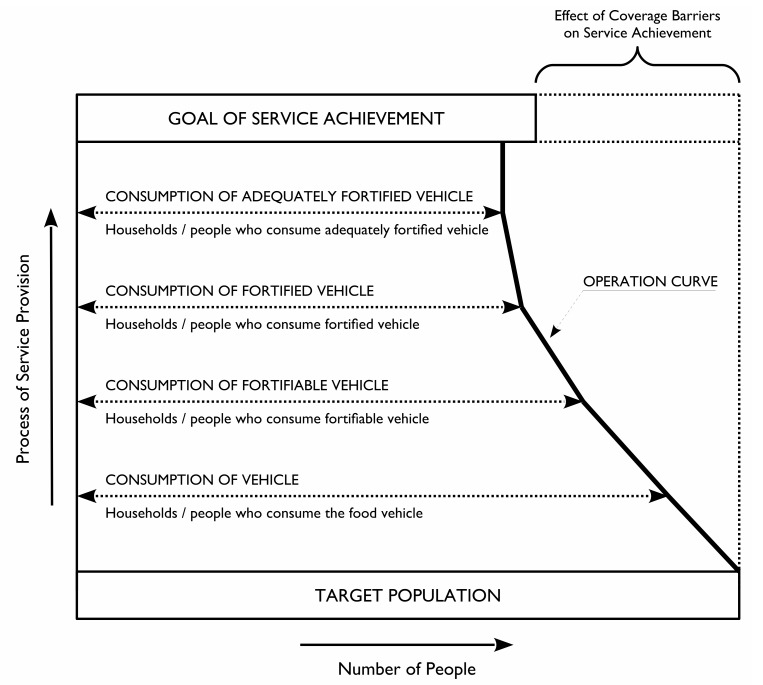
Tanahashi model of coverage measures applied to fortification indicators.

Four measures of coverage were assessed using the “bottlenecks” model of Tanahashi [18] as shown in Fig 2. The Tanashi framework of coverage relies on the identification of sequential stages through which coverage is achieved. Each stage relates to an important condition on the pathway to the provision of a service. A coverage measure is defined and measured for each stage. This is usually the proportion of the target population for whom the condition is met. Coverage of service provision is the product of these proportions [18].

The indicators, expressed as the proportion of sampled households covered, were:

- Consumption of the vehicle: The household consumes the food vehicle.
- Consumption of *fortifiable* vehicle: The food vehicle used by the household is processed by medium to large-scale producers and hence is well suited to large-scale fortification. This definition means that small local mills (i.e. “chakkis.”) or home processing would not be considered fortifiable for the present analyses.
- Consumption of *fortified* vehicle: The vehicle used by the household is fortified.
- Consumption of *adequately fortified* vehicle: The vehicle used by the household is fortified according to agreed standards.

Three summary statistics were calculated for each of the four coverage measures:

- Raw Coverage (RC): The proportion of all households that were covered. This is a measure of overall program performance.
- Met Need (MN): The proportion of households defined as at-risk that were covered. This is a measure of how well the program addresses risk/need.
- Coverage Ratio (CR): The ratio of the coverage in at-risk households (MN) to the coverage in households considered to be not at-risk. The CR ranges between 0 and positive infinity. CR values below 1 indicate poor targeting (i.e., coverage is higher in the not at-risk population than in the at-risk population). CR values above 1 indicate good targeting (i.e., coverage is higher in the at-risk population than in the not at-risk population). A CR of exactly 1 indicates an absence of targeting (i.e., coverage is similar in the at-risk and not at-risk populations).

A well-functioning program is typified by having high RC or having both a high MN and a CR above 1 (i.e., coverage is concentrated in the at-risk populations) [14].

### Determination of fortification status

Fortification status was determined by quantitative laboratory analysis for atta wheat flour and salt and by questionnaire only for edible oil (by linking named brands with brands known to be fortified). For atta wheat flour, specimens were collected during the survey from all households that reported using already milled atta wheat flour that were willing to provide samples. All specimens were shipped to a reference laboratory in Germany and analyzed for total iron content using handheld photometers [19]. To determine the amount of added iron from fortification, non-fortified flour samples were collected from markets during the survey and analyzed to allow an adjustment for intrinsic iron. Atta wheat flour samples were classified as fortified if iron was above 33 parts per million (ppm) and as adequately fortified if iron was above 63 ppm. Salt specimens were also collected during the survey from all households willing to provide samples. All specimens were shipped to a reference laboratory in India and analyzed for total iodine content using titration [20]. Salt samples were classified as fortified if total iodine content was at or above 5 ppm and adequately fortified if total iodine content was at or above 15 ppm.

### Indicators of consumption and coverage

**Fig 3:**
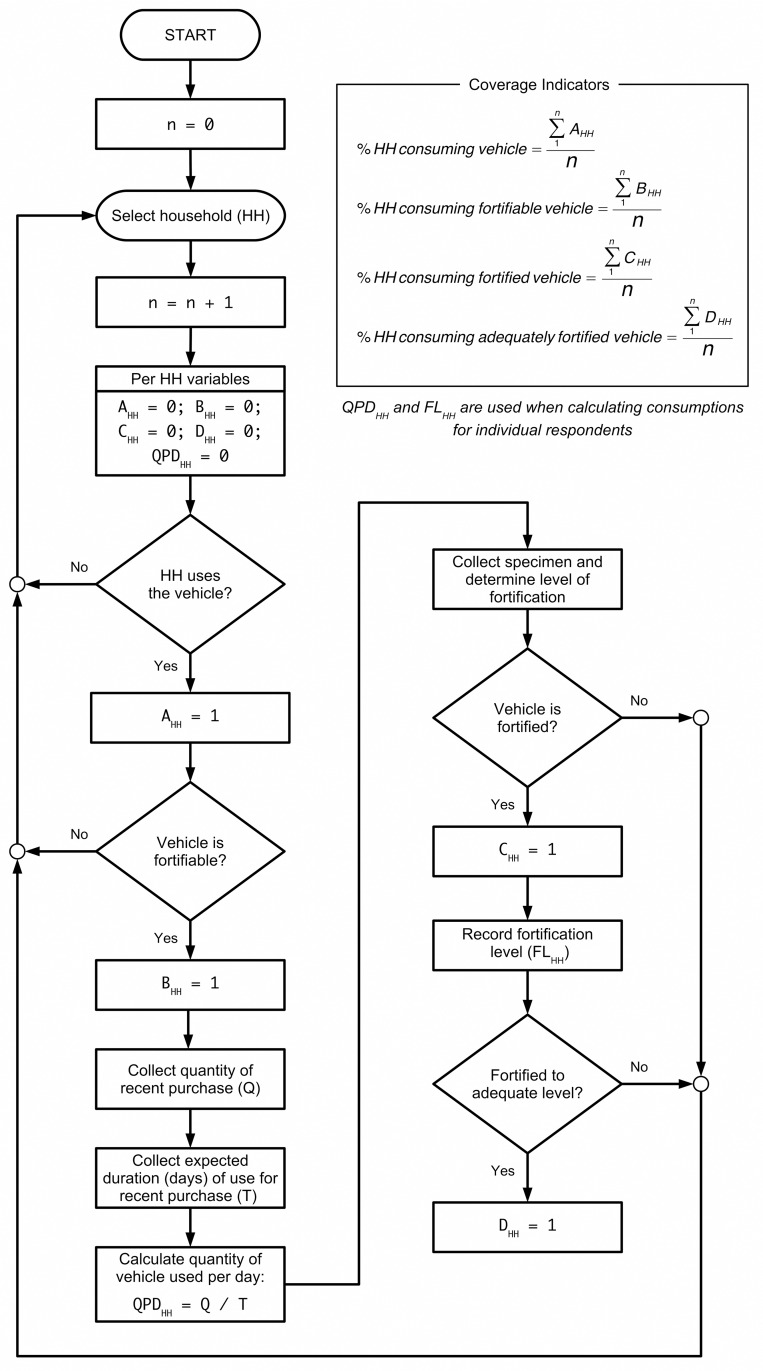
Flowchart showing calculations for coverage indicators.

**Fig 4:**
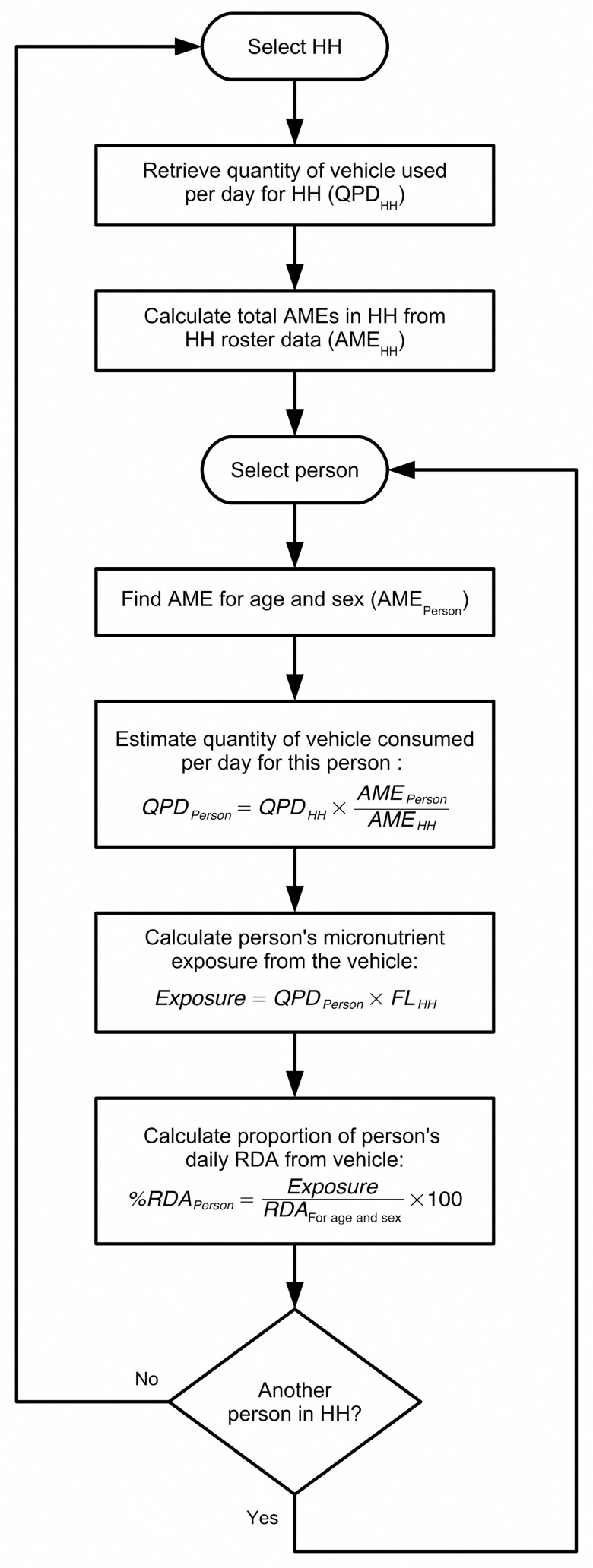
Flowchart showing calculations for consumption indicators. HH = household; QPD = quantity (mass) of vehicle consumed per day (from Fig 3); AME = adult male equivalent; FL = fortification level (from Fig 3); RDA = recommended daily allowance

Fig 3 and Fig 4 show the process (flowcharts and formulas) used to calculate consumption indicators. Total grams of food vehicle consumed in each household for atta wheat flour and edible oil were estimated based on quantities purchased and standardized by day for the household (QPD_HH_). An age and sex-specific adult male equivalent (AME_Person_) was assigned to each member in the household above 6 months of age [21]. These values were then summed to calculate a household adult male equivalent (AME_HH_). This approach assumes individual intakes are proportional to energy needs and that food is rationed accordingly between individuals. The quantity in grams of food vehicle consumed per person per day (QPD_Person_) was estimated, and this was then used to estimate micronutrient exposure levels by multiplying QPD_Person_ by fortification levels (FL_HH_). The micronutrient contributions were computed for atta wheat flour only and expressed as a percentage of the Indian recommended daily allowance (RDA) [22].

### Data analyses

Data were analyzed using the R language for data-analysis and graphics (version 3.2.2) and the R-AnalyticFlow scientific workflow system (version 3.0.1). A blocked weighted bootstrap estimation technique was used [23]. Bootstrap replicates consisted of a set of within-PSU survey samples that were sampled with replacement and with a probability proportion to PSU population size using a *roulette wheel* (also known as *stochastic sampling with replacement*) algorithm [24]. For each bootstrap replicate, a total of *m* PSUs were sampled with replacement (where *m* is the number of PSUs in the survey sample). Observations within selected PSUs were also sampled with replacement with the same within-PSU sample size that was achieved in the survey. A total of *r* = 400 bootstrap replicates were used. The required summary statistic was calculated from each replicate. The resulting estimate consisted of the 2.5th (lower 95% confidence limit), 50th (point estimate), and 97.5th (upper 95% confidence limit) percentiles of the distribution of the statistic across all replicates [25]. This procedure accounts for unequal selection probabilities in the sample design (by applying posterior weighting), as well as for variance lost due to the clustered nature of the sample [23]. The design of the sample allows for results to be mapped [7,8]. Mapping was performed using inverse distance weighting with a power parameter (*p* = 16) large enough to produce a Voronoi diagram [26,27].

## Results

The survey was implemented from December 2013 to February 2014. Table 2 shows the sample sizes achieved and characteristics of the survey population. A total of *n* = 4,627 households were sampled. Basic demographic and education history data were collected for *n* = 29,968 persons using a household roster. Data required for the calculation of the IYCF, MPI, WDDS, and consumption/coverage indicator sets was collected from *n* = 4,627 caregiver / child pairs. The ratio of male to female children in the sample was consistent with data from the most recent statewide census [28].

**Table 2:** Description of the Survey Sample.

Variable		Value
	PSUs/clusters	252
	Households	4,627
Sample size	Persons in households	29,968
	Principal caregivers^a^	4,627^b^
	Children aged 0 to 24 months	4,627^b^
	Caregiver age in years: mean (range)	25.1 (16, 48)
Characteristic	Child age in months: mean (range)	12.2 (0, 24)
	Sex of child: percentage male (95% CI)	51.6% (49.8%, 53.5%)^c^

^a^ A caregiver may have been the child’s biological mother or the person who cared for and gave the child most meals on most days.

^b^ One caregiver / child pair was sampled from each household. If the selected caregiver had more than one eligible child, then the oldest eligible child under 2 years old was selected, as the youngest eligible child would be likely to be aged below 6 months and should be exclusively breastfed.

^c^ Expected percentage of male children aged between 0 and 4 years is 52.1% (2011 census data) [28].

**Fig 5:**
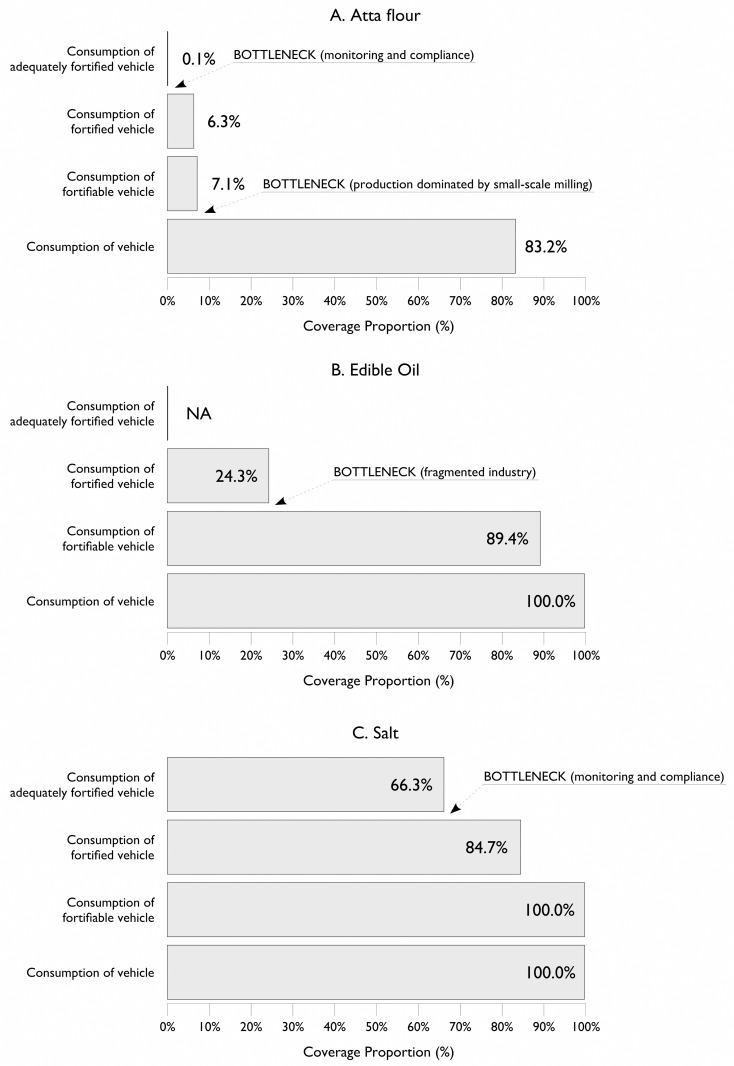
Raw coverages for (A) atta wheat flour, (B) edible oil, and (C) salt.

**Fig 6:**
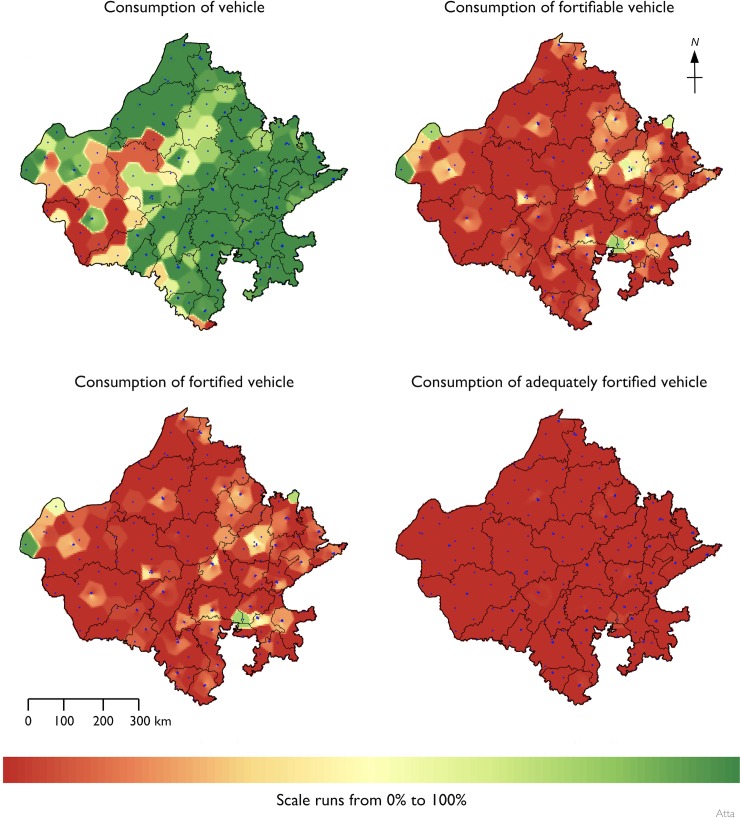
Spatial distribution of raw coverage for atta wheat flour.

**Fig 7:**
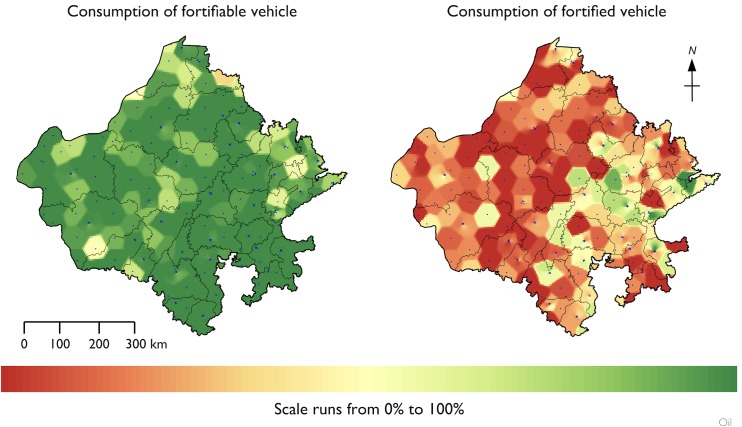
Spatial distribution of raw coverage for edible oil.

**Fig 8:**
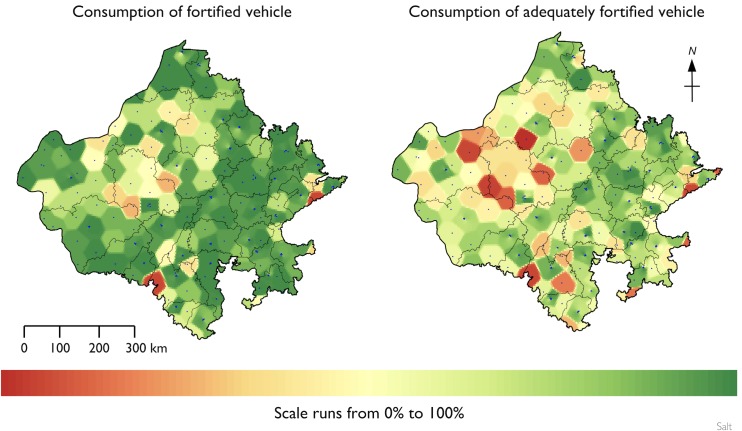
Spatial distribution of raw coverage for salt.

Table 3 presents a summary of atta wheat flour and salt specimens collected and analyzed. Fig 5 presents the RC summaries for the three vehicles. All values are expressed as a percent of the overall survey sample. Summary statistics for risk and coverage for each food vehicle are shown in Table 4. Fewer than 0.4% of households had atta wheat flour from the government PDS system at the time of the survey. The results for the voluntary and the vestigial government programs were merged for the analysis presented in Table 4. All households reported using oil in the home; summary statistics are shown for fortifiable and fortified oil only. All households also reported using salt, and all salt was classified as fortifiable. Summary statistics are shown for fortified and adequately fortified salt only. Table 5 shows the percentage of persons in the survey dataset who received none of their RDA for iron from fortified atta wheat flour and the median percentage of RDA for iron from fortified atta wheat flour for persons consuming fortified atta wheat flour. The spatial distribution of raw coverage for atta wheat flour, edible oil, and salt are shown in Figs 6–8.

**Table 3:** Summary of Atta Wheat Flour and Salt Specimens Collected and Analyzed.

Vehicle	Micronutrient	Specimens	Specimens	Median (interquartile range)
		collected	not analyzed	fortification level (ppm)^a^
Atta wheat flour	Iron	592	0	44.8 (38.2, 50.4)
Salt	Iodine	4,596	4	11.8 (5.2, 17.4)

^a^ Fortification level for atta wheat flour is corrected for intrinsic iron in locally milled unfortified atta wheat flour.

**Table 4:** Summary Statistics for Risk and Coverage for Each Food Vehicle.

			Food vehicle					
			Atta wheat flour		Edible oil^d^		Salt^e^	
Measure^a^	Risk group^b^	% At-risk	%MN^c^	CR^c^	%MN	CR	%MN	CR
		(95% CI)	(95% CI)	(95% CI)	(95% CI)	(95% CI)	(95% CI)	(95% CI)
Consume	Poverty	30.3	66.4	0.73	****	****	****	****
vehicle		(26.9, 33.8)	(59.5, 73.0)	(0.70, 0.80)				
	WDDS	23.5	77.4	0.91	****	****	****	****
		(25.6 21.1)	(71.7, 83.3)	(0.86, 0.97)				
	IYCF	57.4	82.6	0.99	****	****	****	****
		(59.4, 55.5)	(78.5, 86.3)	(0.95, 1.03)				
	Rural	47.3	76.5	0.77	****	****	****	****
		(45.8, 48.7)	(71.3, 81.0)	(0.72, 0.82)				
Fortifiable	Poverty	30.3	5.0	0.63	95.0	1.09	****	****
		(26.9, 33.8)	(3.0, 7.5)	(0.36, 0.94)	(92.8, 96.7)	(1.05, 1.15)		
	WDDS	23.5	7.1	0.99	90.7	1.02	****	****
		(25.6 21.1)	(4.9, 10.1)	(0.70, 1.40)	(87.5, 93.7)	(0.99, 1.05)		
	IYCF	57.4	7.3	1.04	88.8	0.98	****	****
		(59.4, 55.5)	(5.4, 9.3)	(0.78, 1.43)	(86.0, 91.5)	(0.96, 1.01)		
	Rural	47.3	3.0	0.18	86.3	0.89	****	****
		(45.8, 48.7)	(1.8, 4.9)	(0.11, 0.31)	(82.9, 89.7)	(0.86, 0.93)		
Fortified	Poverty	30.3	4.7	0.67	19.7	0.77	75.3	0.85
		(26.9, 33.8)	(3.0, 7.2)	(0.40, 1.03)	(14.6, 25.4)	(0.55, 1.00)	(70.2, 80.0)	(0.80, 0.90)
	WDDS	23.5	5.5	0.84	22.9	0.92	77.4	0.89
		(25.6 21.1)	(3.4, 8.2)	(0.53, 1.21)	(16.6, 29.3)	(0.68, 1.21)	(72.3, 81.9)	(0.83, 0.94)
	IYCF	57.4	6.6	1.06	24.6	1.03	83.5	0.97
		(59.4, 55.5)	(4.8, 8.6)	(0.80, 1.47)	(20.2, 29.4)	(0.86, 1.27)	(80.4, 86.7)	(0.93, 1.00)
	Rural	47.3	2.6	0.18	20.5	0.64	79.5	0.83
		(45.8, 48.7)	(1.4, 4.4)	(0.09, 0.31)	(16.3, 25.2)	(0.49, 0.85)	(76.1, 82.7)	(0.79, 0.86)
Adequately	Poverty	30.3	0.1	0.37	-	-	51.1	0.70
fortified		(26.9, 33.8)	(0.0, 0.4)	(0.00, 6.68)			(46.2, 56.1)	(0.63, 0.76)
	WDDS	23.5	0.0	0.00	-	-	55.6	0.80
		(25.6 21.1)	(0.0, 0.0)	(0.00, 0.00)			(50.2, 61.0)	(0.73, 0.88)
	IYCF	57.4	0.1	1.02	-	-	65.3	0.96
		(59.4, 55.5)	(0.0, 0.4)	(0.0, Inf)			(61.9, 68.4)	(0.90, 1.01)
	Rural	47.3	0.0	0.00	-	-	58.5	0.70
		(45.8, 48.7)	(0.0, 0.1)	(0.00, 0.59)			(55.0, 62.3)	(0.64, 0.75)

^a^ See Fig 2 (Tanahashi coverage model adapted to large-scale fortification programs).

^b^ Poverty = poverty by MPI ≥ 0.33; WDDS = maternal dietary diversity score below median value; IYCF = ICFI score < 6; Rural = rural place of residence.

^c^ %MN = Met Need; CR = Coverage Ratio (see text for details).

^d^ 100% of households consumed oil. Oils specimens were not collected. No analyses for adequate fortification were conducted

^e^ 100% of households salt. All salt was classified as fortifiable.

**Table 5:** Proportion of RDA for Added Iron from Fortified Atta Wheat Flour.

Indicator	Summary	Notes
Percentage with > 0% RDA^a^	6.2%	n = 29,968
	(95% CI = 4.6%; 8.2%)	(all persons in sampled households)
Median % RDA	47.0%	*n* = 2,903
	(95% CI = 43.7%; 50.8%)	(only atta wheat flour consumers)

^a^ RDA values were determined for individual age and sex requirements based on Indian RDA values using the observed fortification level for atta wheat flour corrected for intrinsic iron in locally milled unfortified atta wheat flour.

## Discussion

Results from the present survey provided timely feedback to the Rajasthan fortification program. They also highlight some of the challenges of achieving high coverage through a voluntary food fortification program.

A large percentage (about 83%) of the population in the program area consumes atta wheat flour. This is a key criterion of suitability for large-scale fortification (see Fig 5A and Fig 6). Despite high consumption of the vehicle, most of the population (about 93%) had no exposure to *fortifiable* atta wheat flour (see Fig 5A and Fig 6). This is most likely due to a lack of the requisite consolidation/centralization in the production, processing, and distribution of the vehicle. Most of the surveyed population (about 82%) reported purchasing whole grain and milling either at home or at local small local mills called “chakkis.” This is the principal bottleneck to the program achieving high coverage (see Fig 5A and Fig 6). Among those in the population consuming fortifiable flour (about 6%), almost 90% of the flour specimens analyzed were fortified to some degree. This is a positive finding, indicating that the majority of fortifiable flour is being fortified. However, fortification levels were inadequate, and fewer than 1% of households were consuming adequately fortified atta wheat flour at the time of the survey. This finding identifies a second major bottleneck to the program: monitoring and evaluation of compliance to fortification protocols. The amount of atta wheat flour consumed by the population is large and, among the 6% that consumed fortified atta wheat flour, a median of about 47% of the iron RDA was achieved using values determined for individual age and sex requirements based on Indian RDA values. This is a positive result but suggests that the proposed fortification level did not fit with the consumption patterns for atta wheat flour in the program area (i.e., fortification levels were not appropriate for existing patterns of consumption).

Low overall coverage may be tolerable if coverage is concentrated in the at-risk population. This was not the case for the atta wheat flour program described here (see Table 4). Based on these assessments, the atta wheat flour program failed to target those most likely to be in need of the intervention. Programs that fail to reach at-risk populations are likely to have limited impact. The WHO guidelines on food fortification recognize this and suggest that coverage estimates be made for at-risk populations for all fortification programs [1]. This is an important consideration given that the cessation of the government’s targeted fortified atta wheat flour distribution program will have reduced coverage of fortified atta wheat flour in at-risk populations. The atta wheat flour program coverage results raise questions about the continuation of the program. Considerable capital investments were made to start the program. Considerations should be given as to how these may now be best utilized. One possibility, unless the PDS system is reinstated, would be to direct the fortified flour produced to targeted interventions, such as school and preschool feeding programs.

Almost 90% of edible oil consumed by households in the survey was classified as fortifiable. The CR for consumption of the fortifiable oil was significantly below 1 for households in rural locations but was significantly above 1 for households in poverty. The CR for consumption of the fortified oil was significantly below 1 for households in rural locations. Rajasthan is a large edible oil-producing state with a fragmented industry. It is likely that at least some of the oil classified as fortifiable was produced by small-scale enterprises, and it may not be feasible to include these producers in a large-scale fortification program. This issue requires a more detailed assessment of the oil industry, which was beyond the scope of the survey. It is clear, however, that fragmentation of the industry is a major bottleneck to achieving coverage (see Fig 5B and Fig 7*)*. The finding that the CR for consumption of the fortifiable vehicle was significantly above 1 for households in poverty suggests that there was a missed opportunity for the program to target poorer households.

All households that participated in the survey consumed salt, and most provided a salt sample for analysis. Coverage was high, with almost 85% of households using fortified salt and 66% of households using adequately fortified salt (see Fig 5C and Fig 8). In comparison, a statewide survey undertaken in 2003 reported that 42% of households used adequately iodized salt [29]. Given the widespread and increasing use of fortified salt across the state, salt could be considered as a vehicle for additional micronutrients, such as iron. The feasibility of this would need to be assessed prior to implementation. However, despite the high coverage achieved, the CRs for the consumption of fortified salt and for the consumption of adequately fortified salt were significantly below 1 for households in poverty, households with low dietary diversity, and households in rural locations. It is possible that this program will suffer from a “last mile” problem with regard to meeting the needs of these at-risk populations. One area for further investigation could be to assess whether these populations are served by the PDS system and, if so, this may be a way to further increase the coverage of the salt program.

Planning for effective large-scale fortification programs needs to be informed by detailed investigation of patterns of production, distribution, consumption, and need. Without this due diligence, programs rely on chance to achieve impact. The capital-intensive nature of these programs means that this is a gamble made with high stakes. The main program barriers for atta wheat flour and edible oil identified by the current survey could have been identified prior to the program starting. For example, the Fortification Rapid Assessment Tool (FRAT) was designed to help program managers select suitable vehicles for large-scale fortification [30,31]. A FRAT survey conducted prior to the program starting would have revealed that the atta wheat flour program and, to a lesser extent, the edible oil program would have failed to achieve coverage and impact. The results from this survey show that, in their present form, the voluntary fortification of atta wheat flour and, to a lesser degree, the edible oil program have limited impact. Efforts should be made to restart the distribution of fortified atta wheat flour through the PDS. The salt iodization program was operating well at the time of the survey. Continued attention should be paid to monitoring and compliance of the fortification process, as well as to further reaching poor and rural population groups.

### Strengths and weaknesses

The main strengths of the work reported here are as follows:

- The survey was spatially representative and population representative of the state.
- Standardized and validated indicators were used to assess need and risk.
- Program coverage was linked to risk / need using MN and CR statistics.
- Data were analyzed and presented using the Tanahashi bottleneck analysis, which has been proven useful for a wide range of programming.

The limitations of the work reported here are:

- The method used to determine iron fortification levels in atta wheat flour does not distinguish between intrinsic and added iron. Inconsistencies in flour extraction rates may have resulted in higher levels of intrinsic iron in non-fortified flour samples used to correct the analyses. This may have resulted in the survey underestimating the coverage for consumption of the adequately fortified atta wheat flour.
- Oil specimens were not collected from households. This introduces uncertainty with the interpretation of the fortifiable coverage indicator
- Intake was measured using an indirect approach. Household consumption was apportioned to individuals using the AME approach, which assumes individual intakes are proportional to energy needs and that food is rationed equitably between individuals.
- The survey instruments did not capture foods purchased and consumed outside of the households, such as snacks and restaurant meals.

## Conclusions

Under the right conditions, fortification can reach large segments of the population. To be effective at scale, however, requires consumption of industrially fortifiable foods by the bulk of the population (particularly at-risk populations), as well as strong commitment from industry and government to ensure that the selected food vehicles are adequately fortified. These conditions were not met for the atta wheat flour and edible oil programs in Rajasthan. The salt iodization program achieved high coverage but needs to find ways to improve delivery to rural and poor population groups. Voluntary fortification of atta wheat flour and edible oil lacked the sufficient industry consolidation to cover significant proportions of the population. It is crucial that appropriate delivery channels are utilized to effectively deliver essential micronutrients to at-risk population groups. In terms of fortification, government distribution systems such as the PDS and school feeding programs are likely the best means to do this. In any distribution model, routine monitoring and enforcement are both essential to ensure that fortification protocols are followed.

## Supporting Information

S1 Survey instrument (PDF)

S1 Table (DOCX)
