# Supplementary material for: Household Coverage of Fortified Staple Food Commodities in Rajasthan, India
Source: PLoS One. 2016 Oct 19;11(10):e0163176. doi: 10.1371/journal.pone.0163176 (PMC5070859; doi:10.1371/journal.pone.0163176)
Supplement: S1 Survey instrument — (PDF) [file pone.0163176.s001.pdf]

IIHMR Health and Nutrition survey
आईआईएचएमआर स्वास्थ्य एवं पोषण सर्वेक्षण

Informed Consent: Greetings! I am ___________________________ and I have come on behalf of Indian Institute of Health Management Research (IIHMR) Jaipur. We are carrying out a survey on health and nutrition. This discussion is expected to last approximately 15-20 minutes. Any information given by you will be kept confidential. Your participation in the survey is voluntary. However, we hope that you would take part in the survey since your participation is important. At any point in the survey if you feel that you're not ready to answer the question you can ask me to skip the question. At this point do you want to ask me anything about the survey? (Answer any question and address concerns of the respondent).

TO BE FILLED IN BY INVESTIGATOR

Village/town name: __________________________________

psu Primary sampling unit |__|__|__|
hh Household id |__|__|
iid Investigator id |__|__|

Investigator name: ___________________ Signature: ____________________

Date: __________________

****

FOR OFFICE USE ONLY

sid Supervisor: ___________________ Signature: _____________ |__|__|

feid Field editor: _________________ Signature: _____________ |__|__|

oeif Office editor: ________________ Signature: _____________ |__|__|

d1id Data entry op1: _______________ Signature: _____________ |__|__|

d2id Data entry op2: _______________ Signature: _____________ |__|__|

Remarks:

Name: __________________ Signature: _________________

Fill in the information about the sampled mother and the child:

	Name	Sex (M=1, F=2)	Completed age (years)	Completed age (months)	Ever attended school or college?	Currently attending school or college?	5 or more years of education?
**mother**		__	__ __		__	__	__
**child**		__		__ __			

Who are family members that live with you now or mostly live with you?
Complete one line of the roster for each remaining household member:

	Name	Sex (M=1, F=2)	Completed age (years)	Completed age (months)	**For persons aged ≥ 5 years** Ever attended school or college?	Currently attending school or college?	5 or more years of education?
1		__	__ __	__ __	__	__	__
2		__	__ __	__ __	__	__	__
3		__	__ __	__ __	__	__	__
4		__	__ __	__ __	__	__	__
5		__	__ __	__ __	__	__	__
6		__	__ __	__ __	__	__	__
7		__	__ __	__ __	__	__	__
8		__	__ __	__ __	__	__	__
9		__	__ __	__ __	__	__	__
10		__	__ __	__ __	__	__	__
11		__	__ __	__ __	__	__	__
12		__	__ __	__ __	__	__	__
13		__	__ __	__ __	__	__	__
14		__	__ __	__ __	__	__	__
15		__	__ __	__ __	__	__	__
16		__	__ __	__ __	__	__	__

Check the roster regarding completeness!

re : What is your religion? *Tick one box only!*

Hindu	|__|	re=1
Muslim	|__|	re=2
Sikh	|__|	re=3
Christian	|__|	re=4
Jain	|__|	re=5
Buddhist	|__|	re=6
None	|__|	re=7
Prefer not to say	|__|	re=8
Other	|__|	re=9

MPI (Assets) Data

mpi1 : Does your household have electricity? |__|

mpi2 : What fuels does your household use for cooking?
*(If dirty fuel used then tick **only** for dirty fuel.)*

Electricity
Gas (LPG)
Biogas (Gobar gas)
Kerosene
Solar
} |__| mpi2=1

Coal / lignite
Charcoal
Wood
Straw / shrubs / grass / crop residue
Animal dung
} |__| mpi2=2

mpi3 : Main material of the floor of the dwelling?

*Tick **one box** only ...*

Dirt / earth / sand / dung / mixed	|__|	mpi3=1
Other (tiles, concrete, cement, wood)	|__|	mpi3=2

mpi4 : Does your household have ... ? *Fill in **all** boxes!*

***Prompt** for each item.*

A radio?	|__|	mpi4a
A television?	|__|	mpi4b
A mobile or any other telephone?	|__|	mpi4c
A bicycle or a tricycle?	|__|	mpi4d
A motorcycle, scooter, auto-rikshaw?	|__|	mpi4e
A car, truck, jeep, or tractor?	|__|	mpi4f
A refrigerator?	|__|	mpi4g
A computer / laptop / tablet	|__|	mpi4h
A horse/ox/camel/mule/donkey cart?	|__|	mpi4i

Short Birth History

The purpose of the short birth history (SBH) component is to determine whether any child that was recently born to the respondent has died within the past five years.

Tell the mother :

I will be asking about your recent births.

I am interested in your three most recent births.

I am interested in all children that were born alive.

Ask the following questions :

When was your most recent birth?

Where is this child now?

If **DEAD** then **record the death (YES) in d1** below and **GO TO w1**

Did you have a birth before this most recent one?

If **NO** (no previous birth) then record **NO in d1** below and **GO TO w1**

If **YES**: *Where is this child now?*

If **DEAD** then **record the death (YES) in d1** below and **GO TO w1**

Did you have a birth before that one?

If **NO** (no previous birth) then record **NO in d1** and **GO TO w1**

If **YES**: *Where is this child now?*

If **DEAD** then **record the death (YES) in d1** below and **GO TO w1**

d1 : A recently born child has died : |__|

Water, Sanitation, and Hygiene (WASH)

w1 : What is the **main source** of drinking water for you and your child?

Do not prompt!

*Tick **one box** only!*

Piped water into dwelling / Tap
Piped water into yard / plot / compound
Public tap
Standpipe
Tube-well
Borehole
Protected dug well/spring
Rainwater collection system
Bottled water / sachet water

|__| w1=1

Unprotected dug well/spring
Cart with small tank or drum
Tanker-truck
River or stream
Dam, lake, or pond
Canal or irrigation channel
Other

|__| w1=2

w2 : What do you **usually** do to the water to make it safer to drink?

Do not prompt

Probe: 'Anything else?'

*Tick **one box** only ...*

Boil
Add bleach / chlorine tablet
Use a water filter / gravel / ceramic / sand
Solar disinfection

|__| w2= 1

Strain it through a cloth **only**
Let it stand and settle **only**
Nothing
Don't know
Other

|__| w2= 2

w3 : What kind of toilet facility do members of your household **usually** use?

Do not prompt

*Tick **one box** only ...*

Flush or pour flush into:

- Piped sewer system
- Septic tank
- Pit latrine
- Don't know

Pit latrine with slab
VIP Latrine
Composting toilet

|__| w3=1

Flush or pour flush into:

- Elsewhere (not specified above)

Pit latrine **without** slab
Bucket
Hanging latrine
Bush, field, or no facilities → **tick [YES] w4**

|__| w3=2

w4 : Do you share this facility with other households? |__|

Household Hunger Scale (Food Security)

hh1 : How many times in the last month was there ever no food to eat of any kind in your house because of lack of resources to get food?

Number of times : |__|__|

(RECORD 00 FOR "NONE")

hh2 : How many times in the last month did you go to sleep at night hungry because there was not enough food?

Number of times : |__|__|

(RECORD 00 FOR "NONE")

hh3 : How many times in the last month did you go a whole day and night without eating anything at all because there was not enough food?

Number of times : |__|__|

(RECORD 00 FOR "NONE")

Infant and Young Child Feeding (IYCF)

f1 : Is [NAME OF CHILD] currently breastfed? |__|

f2 : Does [NAME OF CHILD] take any food or drink other than breastmilk?|__|

f3 : How many times was [NAME OF CHILD] fed mashed or pureed food or solid or semi-solid food as a meal or a snack since this time yesterday?

Number of times : |__|__|

(RECORD 00 FOR "NO FOOD")

Mother and Child Dietary Diversity

Since this time yesterday what food did you and [NAME OF CHILD] eat?

Probe: "Did you have any of the following things to eat or drink" for items not mentioned

**Check whether mother and child vegetarian or not to avoid offense in DD10-12*

Tick or cross ALL boxes!

		Mother	Child
**DD01**	Plain water?	|__|	|__|
**DD02**	Tinned, powdered or fresh milk (excluding breast milk), Cares or infant formula such as Cerelac, Lactogen, Mother Dairy, Nan Pro (Nestlé)	|__|	|__|
**DD03**	Tea or infusion, light soup, sweetened or flavoured water, sodas, malt drinks (Horlicks), coffee, janam ghutti, nimou, liquor, beer	|__|	|__|
**DD04**	Any food made from grain such as millet (bajra), wheat, rice, maize, semolina, atta flour, maggi noodles, porridge, jau	|__|	|__|
**DD05**	Any food made from fruits or vegetables that have yellow or orange flesh such as red carrots, kachar, red sweet potatoes, ripe mangoes, ripe papaya, balam kakdi, khadoo, kachri	|__|	|__|
**DD06**	Any dark green leafy vegetables (palak, methi, cholei, sarso, bathua, sejan, spring onion and radish leaves)	|__|	|__|
**DD07**	Any food made from roots or tubers such as arbi, moli, jimmikand (white), white potatoes, white sweet potato, onions, ginger	|__|	|__|
**DD08**	Any food made from lentils, beans, Bengal gram, channa, urad, peas, green gram, horse gram, besan, moong, soya bean, ker, cowpea, sattu, peanut paste, kichari, nuts, or seeds	|__|	|__|
**DD09**	Any other fruits or vegetables (coconut, apple, green mango, banana, pomegranate,lemon, guava, brinjal, tomatoes, beetroot, fali, tinda, kakri, parwal, gilki, peppers, singara, lal sag, lauki, toru, guar, gawanar, kakdi, cauliflower, cabbage, cucumbers, sweet corn, sangri, kudu, bjindi)	|__|	|__|
**DD10**	Liver, kidney, heart, or other organ meats	|__|	|__|
**DD11**	Any meat such as chicken, goat, sheep, teetar, beef, pork	|__|	|__|
**DD12**	Fresh or dried fish, shellfish, or seafood, crabs, frogs, lizards and snakes, grubs	|__|	|__|
**DD13**	Curd, lassi, yoghurt, chaach, cheese, paneer, mawa barfi, shrikhand or other milk products	|__|	|__|
**DD14**	Eggs.	|__|	|__|
**DD15**	Sugary foods such as biscuits, sweets, candies, sugar cane, cakes, and chocolate	|__|	|__|
**DD16**	Any food made with oil, fat, butter, or ghee	|__|	|__|
**DD17**	Red palm oil	|__|	|__|

FLOUR FORTIFICATION COVERAGE

ff1 : Ask the mother:

What types of flour does your household have now? Fill in ***ALL boxes****!*

What is the main flour consumed by your household? Tick ***ONE box*** *only!*
**If two main flours consumed, give preference to atta wheat flour!*

Where do the flours come from? Fill in ***ALL RELEVANT boxes!***

		Have now	Main flour	Where from
**ff1a**	Raj Atta flour	|__|	|__|	|__|
**ff1b**	Atta wheat flour	|__|	|__|	|__|
**ff1c**	Maida wheat flour	|__|	|__|	|__|
**ff1d**	Millet flour	|__|	|__|	|__|
**ff1e**	Maize flour	|__|	|__|	|__|
**ff1f**	Barley flour	|__|	|__|	|__|
**ff1g**	Mixed flour	|__|	|__|	|__|
**ff1h**	Other:___________	|__|	|__|	|__|
**ff1i**	Other:___________	|__|	|__|	|__|

Codes for "where from":

1 = Ground grains at home

2 = Ground grains at chakki

3 = PDS/ration shop

4 = Purchased from market/retailer

5 = Other

If ***NO*** *to* ***Raj*** *atta (have and main) but* ***YES*** *to* ***atta*** *wheat (have* ***OR*** *main)→* ***GO TO ff4***

If ***NO Raj*** *atta* ***AND NO atta*** *wheat flour →* ***GO TO ff10***

If ***YES*** *to* ***Raj*** *atta wheat (have* ***OR*** *main) →* ***GO TO ff2***

ff2 : For the most recent major purchase of Raj atta, how much did your household buy?

Amount in kilos (kg): |__|__|__|

ff3 : *How long does this amount usually last in your household?*

Number of days: |__|__|__|

If **YES** to **atta** (have **OR** main) in ff1b → **GO TO ff4**
If **NO** to atta (main **AND** have) in ff1b → **GO TO ff9 and take a sample**

ff4 : For the most recent major purchase/grinding of atta wheat flour, how was the flour packaged? *Tick **one box** only!*

Ground own, or at chakki	|__|	ff5=1
Don't know	|__|	ff5=2
"Open" flour (any quantity)	|__|	ff5=3
Packaged flour (any quantity)	|__|	ff5=4
Other	|__|	ff5=5

ff5 : For the most recent major purchase/grinding of atta wheat flour, how much did your household buy/grind?

Amount in kilos (kg): |__|__|__|.|__|__|

ff6 : How long does this amount usually last in your household?

Number of days: |__|__|__|

ff7 : Can you tell me the brand? *Tick **one box** only!*

Unbranded / grind at home or at chakki → **GO TO ff10**	|__|	ff8=1
Don't know brand (e.g. if open flour or someone else purchased)	|__|	ff8=2
Lakshmi Bhog	|__|	ff8=3
Aashirvaad	|__|	ff8=4
Shree Bhog	|__|	ff8=5
Swastik	|__|	ff8=6
Balaji	|__|	ff8=7
Other-specify: ________________________	|__|	ff8=8

ff8 : Do you have any of above mentioned flour available right now? |__|

if **YES** → **GO TO ff9**, if **NO** → **GOTO ff10**

ff9 : May I take a small sample? |__|

If YES → *Record the PSU ID and Household ID below and on the sachet*

ff9sn Sample number: |__|__|__|/|__|__|
PSU ID HH ID

ff10 : Ask the mother:

In the last 7 days how many times did you and [NAME OF CHILD] eat products made from maida, such as [food item]?

If frequency = 0, don't ask the portion size!

Usually how much of [food item] did you and [NAME OF CHILD] eat at one sitting? Show pictures!

	**Food item**	**Mother**		**Child**	
		Frequency (# times)	Portion size	Frequency (# times)	Portion size
**ff10a**	Biscuits	|__|__|	|__|__|	|__|__|	|__|__|
**ff10b**	Toast / Rusk	|__|__|	|__|__|	|__|__|	|__|__|
**ff10c**	Bread	|__|__|	|__|__|	|__|__|	|__|__|
**ff10d**	Samosa	|__|__|	|__|__|	|__|__|	|__|__|
**ff10e**	Maggi Noodles	|__|__|	|__|__|	|__|__|	|__|__|
**ff10f**	Jalebi	|__|__|	|__|__|	|__|__|	|__|__|
**ff10g**	Fen	|__|__|	|__|__|	|__|__|	|__|__|
**ff10h**	Matri	|__|__|	|__|__|	|__|__|	|__|__|
**ff10i**	Kachori	|__|__|	|__|__|	|__|__|	|__|__|
**ff10j**	Shakar Pare	|__|__|	|__|__|	|__|__|	|__|__|
**ff10k**	Naan	|__|__|	|__|__|	|__|__|	|__|__|
**ff10l**	Pav	|__|__|	|__|__|	|__|__|	|__|__|
**ff10m**	Kulcha	|__|__|	|__|__|	|__|__|	|__|__|
**ff10n**	Patties	|__|__|	|__|__|	|__|__|	|__|__|
**ff10o**	Bun	|__|__|	|__|__|	|__|__|	|__|__|
**ff10p**	Pizza	|__|__|	|__|__|	|__|__|	|__|__|

Probe: Did you eat anything else made from maida (e.g. bathura, balushai, gujiya etc)?

Usually how much of the [food item] did you and [NAME OF CHILD] eat at one sitting?

	**Food item**	**Mother**	**Child**		**Food item**
**ff10q**	Other: ______________________	|__|__|	|__|__|	|__|__|	|__|__|
**ff10r**	Other:________________________	|__|__|	|__|__|	|__|__|	|__|__|

OIL FORTIFICATION COVERAGE

of1 : Ask the mother:

What types of edible oils does your household have now? Fill in ***ALL* boxes***!*

What is the main edible oil consumed by your household? *Tick* ***ONE* box** *only!*
If using Desi ghee + other oil, give priority to other oil as the main oil

Where did the oils come from? Fill in ***ALL relevant* boxes***!*

	Oil type	Have now	Main oil	Where from	
					**Codes for "where from":**<br>1 = Produced at home<br>2 = Purchased from market/retailer<br>3 = PDS/ration shop<br>4 = Other
**of1a**	Soya-bean oil	|__|	|__|	|__|	
**of1b**	Mustard seed oil	|__|	|__|	|__|	
**of1c**	Sesame seed oil	|__|	|__|	|__|	
**of1d**	Groundnut oil	|__|	|__|	|__|	
**of1e**	Sunflower oil	|__|	|__|	|__|	
**of1f**	Rice-bran oil	|__|	|__|	|__|	
**of1g**	Vegetable blend oil	|__|	|__|	|__|	
**of1h**	Palm oil	|__|	|__|	|__|	
**of1i**	Vanaspati Ghee/Dalda	|__|	|__|	|__|	**→ if Desi Ghee = main oil then move to mi1**
**of1j**	Desi Ghee	|__|	|__|	|__|	
**of1k**	Don't know	|__|	|__|	|__|	
**of1l**	Other___________	|__|	|__|	|__|	
**of1m**	Other___________	|__|	|__|	|__|	

of2 : For the most recent major purchase of [**main oil type**], how was the oil packaged? *Tick* ***one box*** *only!*

Produce oil at home	|__|	of2=1
Don't know	|__|	of2=2
"Open" oil(any quantity)	|__|	of2=3
User pack of 5L/kg or less	|__|	of2=4
User pack of 10L/kg	|__|	of2=5
Tin of 15L/kg or bigger	|__|	of2=6
Other: _________________	|__|	of2=7

OIL FORTIFICATION COVERAGE

of3 : For the most recent major purchase/home production of [**main oil type**], how much did your household buy/produce?

Quantity: |__|__|__|.|__|__|

Units: |__|
L=1, kg=2

of4 : How long does this amount usually last in your household?

Number of days: |__|__|__|

of5 : Can you tell me the brand?

Unbranded / made at home	|__|	of5=1
Don't know (e.g. if open or someone else purchased it)	|__|	of5=2
Chambal	|__|	of5=3
Fortune	|__|	of5=4
Mahakosh	|__|	of5=5
Parampara	|__|	of5=6
Engine	|__|	of5=7
Other: ___________________	|__|	of5=8

MILK

mi1 : Where does your household usually get milk?

Home production	|__|	mi1=1
Market/retailer	|__|	mi1=2
SARAS	|__|	mi1=3
Lotus	|__|	mi1=4
Amul	|__|	mi1=5
Divya	|__|	mi1=6
Onest	|__|	mi1=7
Mother dairy	|__|	mi1=8
Gift/Relief	|__|	mi1=9
Don't know	|__|	mi1=10
Other	|__|	mi1=11

SALT FORTIFICATION COVERAGE

sf1 : The last time your household purchased salt, how was it packaged?

Tick one box only!

Packed salt	|__|	sf1=1
Open salt	|__|	sf1=2
Don't know	|__|	sf1=3

sf2 : Can you tell me the brand? *Tick one box only!*

Tata	|__|	sf2=1
Bhaskar	|__|	sf2=2
Surya	|__|	sf2=3
Saffola	|__|	sf2=4
Captain Cook	|__|	sf2=5
Don't know	|__|	sf2=6
Other:______________________	|__|	sf2=7

sf3 : May I take a small sample? |__|

If YES → *Record the PSU ID and Household ID below and on the sachet*

sf3sn Sample number: |__|__|__|/|__|__|

PSU ID HH ID

Mother and Child Health and Nutrition Data

	Mother	Child
**VAS**	NOT APPLICABLE	**SHOW:** vitamin A syrup<br>Took in past 6 months? |__|
**FEFOL**	**SHOW:** Iron tablet<br>Took during pregnancy? |__| Number received: |__|__|__| Number used: |__|__|__| Enter: 777 if don't know	NOT APPLICABLE
**OEDEMA**	NOT TAKEN	Oedema present |__| If o**edema YES → Refer!**<br>*Enter 8 if refused*
**BCG**	NOT TAKEN	BCG scar |__| ***Note:*** Check left arm for scar<br>*Enter 8 if refused*
**MUAC**	***Note:*** Take MUAC on the **left** arm<br>|__|__|__| mm<br>Enter:<br>777 if MUAC too big<br>888 if mother refused<br><br>How was MUAC taken?<br>Bare |__| muact=1<br>Clothing (thin) |__| muact=2<br>Clothing thick |__| muact=3<br>Bangle |__| muact=4<br><br>If mother **MUAC < 210 mm → REFER!**	***Note:*** Take MUAC on either arm<br>|__|__|__| mm<br>*Enter 888 if refused*<br>Refer child to health facility if:<br>**<6 months** and MUAC **< 110 mm**<br>**>6 months** and MUAC **< 115 mm**
**HC**	NOT APPLICABLE	Mother child protection card |__|

****** CHECK THE QUESTIONNAIRE & THANK THE MOTHER!******
